# Book Reviews

**Published:** 1887-12

**Authors:** 


ANATOMY, DESCRIPTIVE AND SURGICAL. By Henry Gray, F. R. S. Drawings by H. V. Carter, M. D., with additional drawings in later editions. Edited by T. Pickering Pick. A new American, from the eleventh English edition. Thoroughly revised and re-edited, with additions by William W. Keen, M. D. To which is added LANDMARKS, MEDICAL AND SURGICAL. By Luther Holden, F. R. C. S. Pp. 1064. Philadelphia: Lea Brothers & Company. 1887. Chicago: A. C. McClurg & Company.

The publishers of this work very properly say that "any marked change in a text-book so universally in use as Gray's Anatomy becomes a matter of no inconsiderable interest to the profession; and we do not, therefore, feel that an apology is necessary in handing you herewith a transcript of the title-page and prefaces of the new edition of that classic work."

"While thoroughly revised, the new edition possesses a marked feature in the use of colors in illustration wherever they can be of service in enabling the eye to more readily follow the details of anatomical construction."

The book comes to us as an old friend in a new garb, that is in keeping with the advanced ideas of the present age.

The names of the eminent medical men given on the title-page are a guaranty of good work, well done, and the fact that the American publishers are Messrs. Lea Brothers & Company warranted the expectation that it would be issued in a manner worthy of so deserving a book, and that expectation has been fully realized.

"In this (eleventh) edition considerable alterations have been made. The introduction of former editions has been incorporated in the body of the work, for the most part in the form of two sections—one on General Anatomy, the other on Development. These have been entirely rewritten, in order to keep pace with the ever-increasing activity of research in these branches of the science of Anatomy. Some portions of the Introduction have, however, been removed from their former position, and introduced in their appropriate places in other parts of the work; as, for instance, the minute structure of the spinal cord, which has been inserted, with the general description of this part, in the section devoted to the Nervous System.

The whole of the work has undergone a careful revision."

"In the section on Arthrology the movement or movements permitted in each joint have been carefully revised, and the muscles by which these movements are effected have been given. In the section on Myology the action of each muscle or group of muscles has been carefully considered, and many alterations and corrections made. The anomalous muscles mentioned in former editions have been excluded. It was thought that, interesting as these anomalous conditions may be to the scientific anatomist, they were scarcely necessary, and were to a certain extent out of place in a text-book intended for the use of the student of Anatomy. The other parts of the work, and especially that devoted to Microscopic Anatomy, have been carefully revised, and some alterations in arrangement and detail have been introduced.

The whole of the arteries, veins and nerves in the woodcuts have been colored, and it is hoped that this will give additional clearness to the illustrations and enhance the value of the work. In the section of Osteology the dotted lines showing the attachment of the muscles have also been colored."

The American edition is published both with and without colors. In this American edition one hundred and thirteen new engravings have been added, of which, it is said, many are original, which adds to the interest of the book.

"Among these new illustrations are many of the most obvious utility, such as a series of sections through important joints; a series of frozen sections through the trunk, the extremities and the female pelvis; cuts illustrating the histology of various tissues," etc., etc., thus making very substantial additions to a work that was already too well known, and too fully appreciated, to need other commendation than the fact of its having been a favorite text-book for more than a quarter of a century. In its present form it will be welcomed by the whole medical profession of this country.

DISEASES OF THE FEMALE MAMMARY GLANDS, by Th. Billroyth, M. D., of Vienna; and NEW GROWTHS OF THE UTERUS, by A. Gusserow, M. D., of Berlin, Illustrated. These two works constitute Vol. I X, of the "*Cyclopædia of Obstetrics and Gynæcology*," issued monthly during 1887. New York: William Wood & Company; Chicago: W. F. Keener.

One hundred and fifty-eight of the four hundred and twenty-six pages, which comprise this volume, are written by the distinguished professor of surgery at Vienna. The subject is treated in the clear and complete style which characterizes the author's lectures in surgery.

The work of the Berlin obstetrician suffers by comparison, but is nevertheless admirably done, and the volume, as a whole, fully maintains the reputation established by the earlier numbers of the series.

DISEASES OF THE FEMALE URETHRA AND BLADDER, by F. Winckel, M. D., of the Royal University, Munich; and DISEASES OF THE VAGINA, by A. Breisky, M. D., of the Royal University, Vienna. Edited by Egbert H. Grandin, M. D., of New York. These two treatises constitute Vol.X. of "The Cyclopædia of Obstetrics and Gynecology," issued monthly during 1887. New York: William Wood & Company; Chicago: W. T. Keener.

The authors of this treatise are both popular clinical teachers. We are accustomed to the saying, that whatever the Germans undertake they do thoroughly, and this volume is not an exception to that rule. The size of the book, 393 pages, about equally divided between the two subjects, render the treatment necessarily encyclopædic, as the title of the series indicates The illustrations are numerous (97) and well chosen. They are very much more carefully engraved and printed than those which appeared in the early numbers of the Cyclopædia, and are fairly satisfactory.

"A Handbook of General and Operative Gynæcology." Vol. I. By Dr. A. Hegar (University of Freiburg) and Dr. R. Kattenbach (University of Giessen). In two volumes. This is also Vol. VI. of "A Cyclopædia of Obstetrics and Gynæcology," issued monthly during 1887. New York: William Wood & Company; Chicago: W. T. Keener.

The most important part of this volume, that upon Ovariotomy, is written by the professor at Giessen. He places Keith and Tait at the head of the list of ovariotomists, and makes an earnest attempt to account for the great success of the latter without the use of antiseptics. The conclusion arrived at by the author is that practically an aseptic condition is secured by the care which the operator mentioned takes to avoid sources of infection. Small incisions and rapidity in operating do not receive the credit, in explaining Tait's success, which is usually accorded them. It goes without saying that the rest of the book is well written.

"A Handbook of General and Operative Gynæcology." Vol. II. By Dr. A. Hegar, and Dr. R. Kattenbach. In two volumes. Volume VI. of "A Cyclopædia of Obstetrics and Gynæcology," issued monthly during 1887.

This concluding volume of the handbook treats of operations on the tubes, uterus, broad ligaments, round ligaments and vagina, operations in urinary fistulae, prolapse operations, and operations upon the vulva and perinæum.

As in the first volume, most of this is written by the professor at Giessen. The work of both of the authors is characterized by the usual German thoroughness and conscientiousness, and the two volumes combined make a very desirable addition to the literature of the subject.

